# Taxonomy Identifiers (TaxId) for Biodiversity Genomics: a guide to getting TaxId for submission of data to public databases

**DOI:** 10.12688/wellcomeopenres.22949.1

**Published:** 2024-10-15

**Authors:** Mark Blaxter, Joana Pauperio, Conrad Schoch, Kerstin Howe

**Affiliations:** 1 Tree of Life, Wellcome Sanger Institute, Hinxton, England, UK; 2 European Nucleotide Archive, European Bioinformatics Institute, Hinxton, England, UK; 3 National Center for Biotechnology Information, National Library of Medicine, Bethesda, Maryland, USA

**Keywords:** taxonomic workflows, biodiversity genomics, ENA REST API, species identification, Earth BioGenome Project

## Abstract

Biodiversity genomics critically depends on correct taxonomic identification of the sample from which data are derived. Tracking of that taxonomic information through systems that archive data and report on genome sequencing efforts. For submission of data to the International Nucleotide Sequence Database Collaboration (INSDC) databases (DNA DataBank of Japan [DDBJ], European Nucleotide Archive [ENA] and National Center for Biotechnology Information [NCBI]), samples and data derived from them must be assigned a species-level NCBI Taxonomy taxonomic identifier (TaxId, sometimes referred to as taxId or txid). We thus need to be able to identify the TaxId for a target species efficiently. Because the NCBI Taxonomy does not include all known species and cannot preemptively represent unknown taxa, we also need an efficient process for generating new TaxIds for species not yet listed. This document provides workflows for different kinds of TaxId acquisition scenarios and was created to guide users in these processes. Although developed for European projects such as Darwin Tree of Life and the European Reference Genome Atlas, the workflows are universally applicable and describe the use of ENA in resolving taxonomic issues.

Too Long: Didn't Read (TL;DR):

- Use the ENA REST API programmatically to retrieve TaxIds for target species and confirm that sequence data can be submitted to those TaxIds.
- Use the NCBI Web interface to NCBI Taxonomy to identify potential homotypic synonyms.
- Request a new TaxId from ENA for a species not yet in NCBI Taxonomy, and for species-like entries for which the full Linnaean binomen is not determined (see https://ena-docs.readthedocs.io/en/latest/faq/taxonomy_requests.html#creating-taxon-requests).
- Discuss directly with the NCBI Taxonomy curators or the curators at ENA and NCBI whenever you think there is an opportunity to improve their database.

## Plain language summary

Biodiversity genomics relies on correctly identifying the species of each sample and making sure that information is tracked through systems that store data and report on genome sequencing. When submitting data to major international databases (like DDBJ, ENA, and NCBI), each sample must be given a species-level identifier (called a TaxId). We need a quick way to find the right TaxId for each species. Since the NCBI Taxonomy doesn’t include every known species and can’t represent unknown ones, we also need an efficient process for creating new TaxIds for species not already listed. This document outlines workflows for different situations where a TaxId is needed. Although it was developed for European projects like Darwin Tree of Life and the European Reference Genome Atlas, the approach can be used by anyone.

## Acronyms

TL;DR                    too long; didn’t read

TaxId, taxId, txid   Taxonomic Identifier

ENA                       European Nucleotide Archive

NCBI                      National Center for Biotechnology Information

INSDC                    International Nucleotide Sequence Database Collaboration (ENA, NCBI, DDBJ)

REST                      Representational State Transfer

API                         Application Programming Interface

ToLID                     Tree of Life IDentifier

GoaT                      Genomes on a Tree, metadata presentation system

## Convention

In describing the taxonomic hierarchy, we use “below” or “down” to indicate a more detailed level of classification, and “above” or “higher” to indicate a broader level of classification.

## Introduction

The Earth BioGenome Project (EBP) has presented a visionary goal: sequencing all Earth’s named eukaryotic biodiversity over the next decade ^1^. This call to action has initiated the growth of numerous biodiversity genomics projects ^2– 8^. Each project aims to use modern genomics technology at scale to produce reference genome assemblies of unprecedented quality and quantity ^9^. The EBP strongly encourages affiliated projects to submit their data to open databases, so that the world can benefit from the biological insights derived from these efforts.

To best organise these data, a coherent and consistent taxonomic system is essential. A shared taxonomy of the species being sequenced not only allows projects to coordinate and combine their work and provides a framework for analysis and emergent understanding ^10^. The Linnean nomenclature system and the associated Zoological ( https://www.iczn.org/the-code/the-code-online/) and Algal, Fungal and Plant ( https://www.iapt-taxon.org/nomen/main.php) codes provide a complete solution to the naming of species, and the publication of detailed morphological and molecular diagnostics allow researchers to confirm the species attribution of each specimen sampled for genome sequencing. To organise and provide better access to its data, the International Nucleotide Sequence Database Collaboration (INSDC https://www.insdc.org/; including the National Center for Biotechnology Information [NCBI] ^11^, the European Nucleotide Archive [ENA] ^12^ and the DNA Database of Japan [DDBJ] ^13^) has adopted a well-curated taxonomic system for animals, plants, fungi, and protists. This NCBI Taxonomy ^14, 15^ aggregates traditional taxonomic data, and gives a unique numeric identifier, the TaxId, to every node in the tree of life. Unique, stable TaxIDs are available for every level of taxonomy, from Kingdom to subspecies or strain. New TaxIDs are created when data from species new to the NCBI Taxonomy is published, and synonyms and taxonomic revision are explicitly dealt with by merging and deprecating TaxId nodes. Using a TaxId, a researcher can quickly identify both the place of the taxon on the tree and select all DNA or protein sequences that derive from that taxon, or its descendants. We recommend that interested users read the formal description of NCBI Taxonomy ^15^.

The growth of biodiversity genomics projects has increased the rate of sequencing of species never before represented in the INSDC databases. This wealth of new-to-INSDC species means that researchers are in the position of having to request new TaxIds more frequently than ever before. Here, we provide a “how-to guide” to help researchers identify the correct TaxId for their species of interest. We also offer guidance on how to request new TaxIds for species that have not been sequenced before.

## What is a TaxId and what sort of TaxId do I need?

**TL;DR: You need the TaxId of a species (or a name below species level) to submit sequence data**

**Figure 1.**
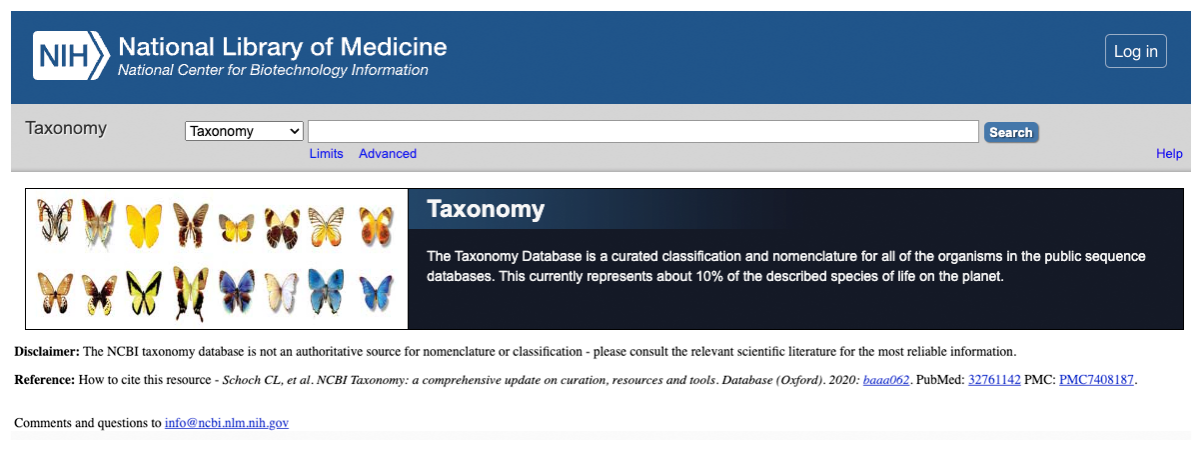
Web access to NCBI Taxonomy. The web browser view of the NCBI Taxonomy at https://www.ncbi.nlm.nih.gov/taxonomy.

**Figure 2.**
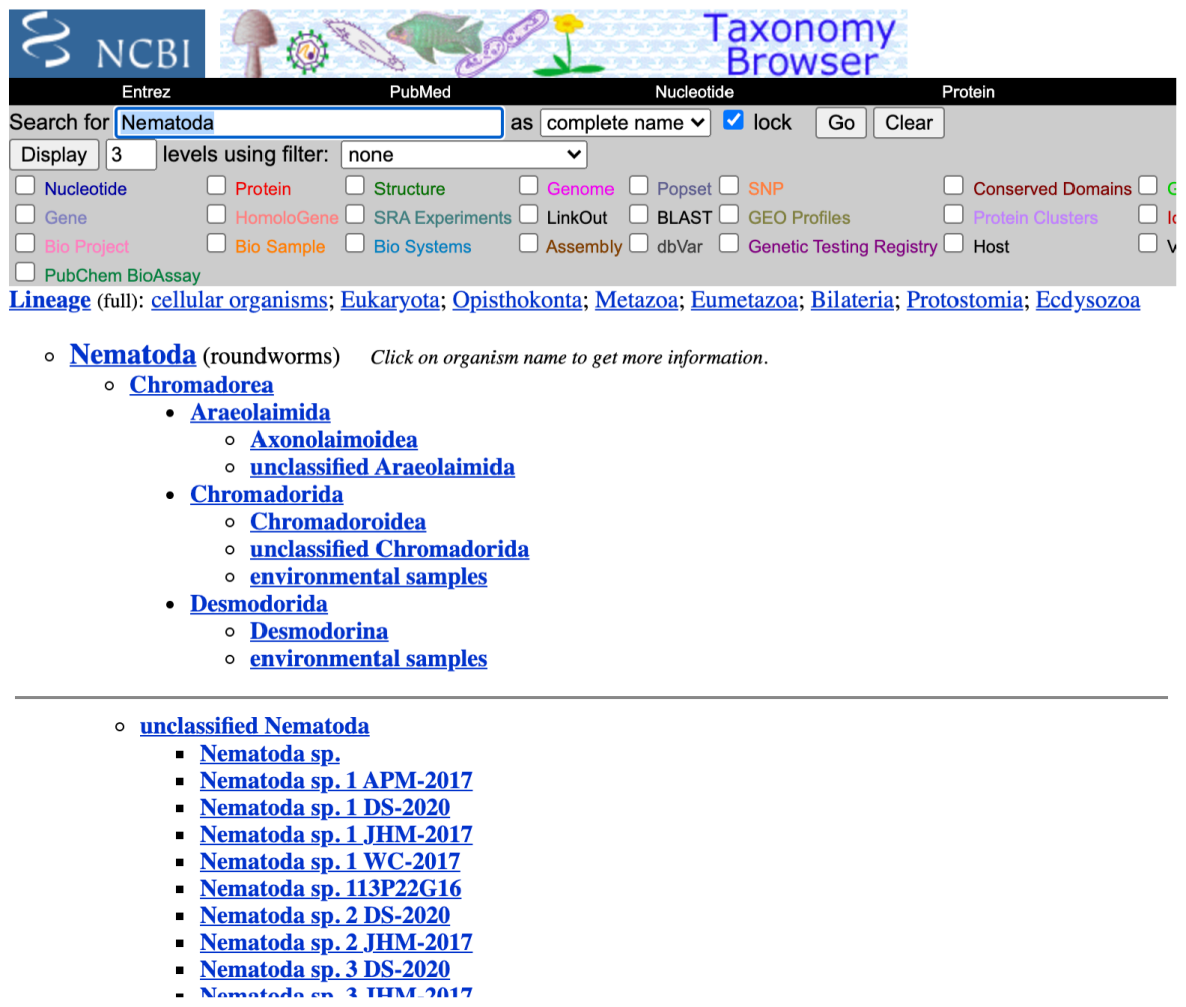
Submittable-to TaxId at all levels. Taxonomy Browser view of the entry for Nematoda, TaxId 6231 ( https://www.ncbi.nlm.nih.gov/datasets/taxonomy/tree/?taxon=6231). The Image has been edited to show the “unclassified Nematoda” taxa.

The TaxId is a numeric identifier corresponding to a node in the National Center for Biotechnology Information (NCBI) Taxonomy taxonomic tree ( Figure 1). In genomic science, we are most interested in the TaxIds to which we can submit sequence data. TaxIds or taxa to which sequence data can be directly submitted are species and formal infraspecies ranks available under their relevant code of nomenclature ( *subspecies*, *variety*, *forma*, etc.). This includes samples with uncertain species affiliation, where a submittable TaxId is generated for objects that link directly to “portmanteau” unclassified taxa. As seen below, a sample with uncertain species affiliation can be indicated as either “<closest confident classification> sp.” e.g. “Nematoda sp.”, “Chromadorea sp.”, etc. ( Figure 2). These choices should be conservative and always include well-formatted voucher information so that individual records can be updated when more precise taxonomic information is available.

An “ *unclassified*” taxon is available in the first level above each of the main branches of cellular life (“ *unclassified bacteria*”, *“unclassified archaea*” and “ *unclassified eukaryotes*”), for cases where there is no identifiable species or above taxon that can be used as a hook on which to hang the data (see Figure 2). Sometimes samples may consist of a macrobiont that is the target of sequencing and one or more microbionts that are mutualist, commensal or parasitic/pathogenic cobionts of the target species. The genomes of these cobionts may inevitably be sequenced and assembled alongside that of the host, target organism. In these cases, the raw data should be submitted under the taxon name and TaxId of the identifiable, species-level taxon of the target organism. The disentangled genomes of the cobionts can be submitted under TaxIds that refer to their individual identities. For example, for a coral sample, raw data and cnidarian host assembly would be submitted under the cnidarian host TaxId, and the symbiotic *Symbiodinium* algal genome assembly submitted under a *Symbiodinium* TaxId. The “ *environmental samples*” taxon found below each main node of cellular life should be used for metagenomic and metabarcoding samples.

### The NCBI Taxonomy

The NCBI Taxonomy can be browsed and queried at https://www.ncbi.nlm.nih.gov/taxonomy ( Figure 1). TaxIds are issued for all nodes in the tree, including sub-species and strain levels. The taxonomy is not fully resolved (so there are many polytomies) and is based on the NCBI Taxonomy curators’ best estimates. They are advised by taxonomic experts in different groups but make no promise or assertion that their taxonomy is the best or the correct one – it is “merely” the one used by the INSDC partner databases (DDBJ, ENA and NCBI) to organise their data:

*“The NCBI taxonomy database is not an authoritative source for nomenclature or classification* – *please consult the relevant scientific literature for the most reliable information.”*

The NCBI Taxonomy curators are engaged and invested in making the taxonomy “correct”, or as correct as it can be, and they welcome corrections and clarifications.

New TaxIds are minted by the INSDC partners in collaboration with NCBI Taxonomy. To mint a new TaxId for a species, (or a sample with uncertain species diagnosis, such as “ *Caenorhabditis* sp. 2”, for which a full Linnaean binomen is not yet available), the curators need to know that you expect to submit data against that TaxId.

The taxonomic system curated by the team at NCBI is imported to the ENA directly. There is a real-time delay in updating the ENA representation, and so new taxa and edits to the existing taxonomy are available in the ENA usually about 48 hours after they have been published in the NCBI Taxonomy. While a newly minted taxon can be retrieved through the programmatic tools (the Representational State Transfer Application Programming Interface or REST API) as soon as it is created, a newly minted taxon is only viewable in the NCBI web browser once it has sequence data associated with it.

TaxIds and the taxonomic names they represent can be revised and deprecated. Revision might be where a “good” species name is revised formally by the community, replacing it for instance with a senior synonym. If a taxon in NCBI Taxonomy is found to be a *homotypic* junior synonym to another senior taxon, it is merged, and any search for it returns the senior synonym. If a taxon is declared and later falls into disuse (for example, a genus name that is superseded by a revised allocation, or a deeper node that is found to be paraphyletic), it is deprecated.

If you think that the taxonomy used by NCBI Taxonomy is in error, or that a species name used has been superseded by a new binomen, the curators are *always* pleased to hear from you, and to work with you and others in the taxonomic community to improve their database. Very rarely, the NCBI Taxonomy and ENA web presentations can differ in what is regarded as the senior synonym, or sometimes accept two synonyms in one namespace as distinct species in the other. While this is usually likely to be due to delays in updating, please report any such issues to both NCBI Taxonomy and ENA.

## Finding out if my species has a TaxId

**TL;DR: Use the ENA or NCBI REST APIs (see below)**

Many processes in biodiversity genomics require TaxId, most critically the submission of raw and assembled data to INSDC databases.

To find out if your target species has a TaxId you can:

- Do single queries in the NCBI interface to TaxonomyDB ( https://www.ncbi.nlm.nih.gov/taxonomy)
- Do bulk queries in the NCBI interface to TaxonomyDB ( https://www.ncbi.nlm.nih.gov/Taxonomy/TaxIdentifier/tax_identifier.cgi)
- Do single or bulk queries using the NCBI API (see below)
- Do single queries in the ENA interface to TaxonomyDB ( https://www.ebi.ac.uk/ena/browser/search)
- Do single or bulk queries using the ENA API (see below).

## TaxId currency

To permit submission of data associated with species new to NCBI Taxonomy, NCBI Taxonomy will create new entries in their database on request. ENA has a process integrated with NCBI Taxonomy to request TaxIds as needed. ENA exchanges data with NCBI Taxonomy once (nightly) per 24 hrs, and thus the ENA and NCBI databases can also differ by the taxa for which new TaxIds have been minted in the previous 48 hrs.

Genomes on a Tree ( https://goat.genomehubs.org/) ^16^ also represents the NCBI Taxonomy TaxIds for all species in INSDC, and can be queried using the GoaT command line interface (GoaT-CLI,) or on the web. GoaT may lag behind the NCBI Taxonomy or ENA representation of NCBI Taxonomy by a matter of hours or days.

## Querying NCBI Taxonomy through the web

**Figure 3.**
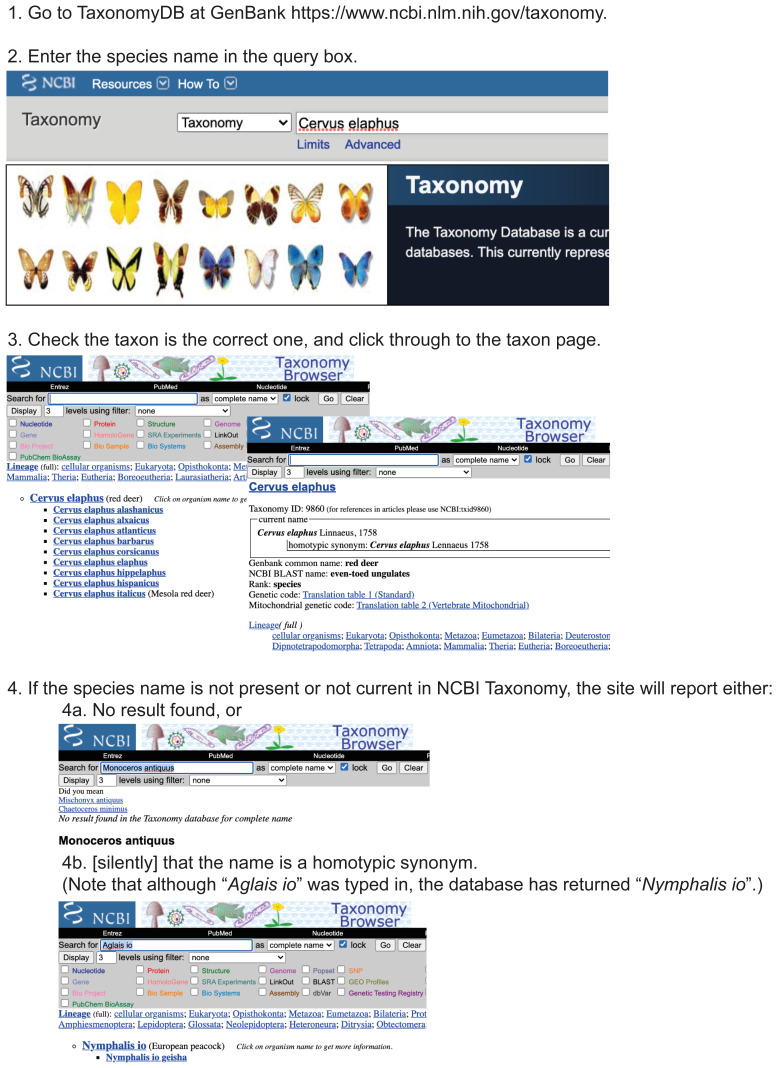
Using the NCBI Taxonomy web interface for single queries. Workflow illustrating the use of the NCBI Taxonomy web interface for a single-name query through https://www.ncbi.nlm.nih.gov/taxonomy.

**Figure 4.**
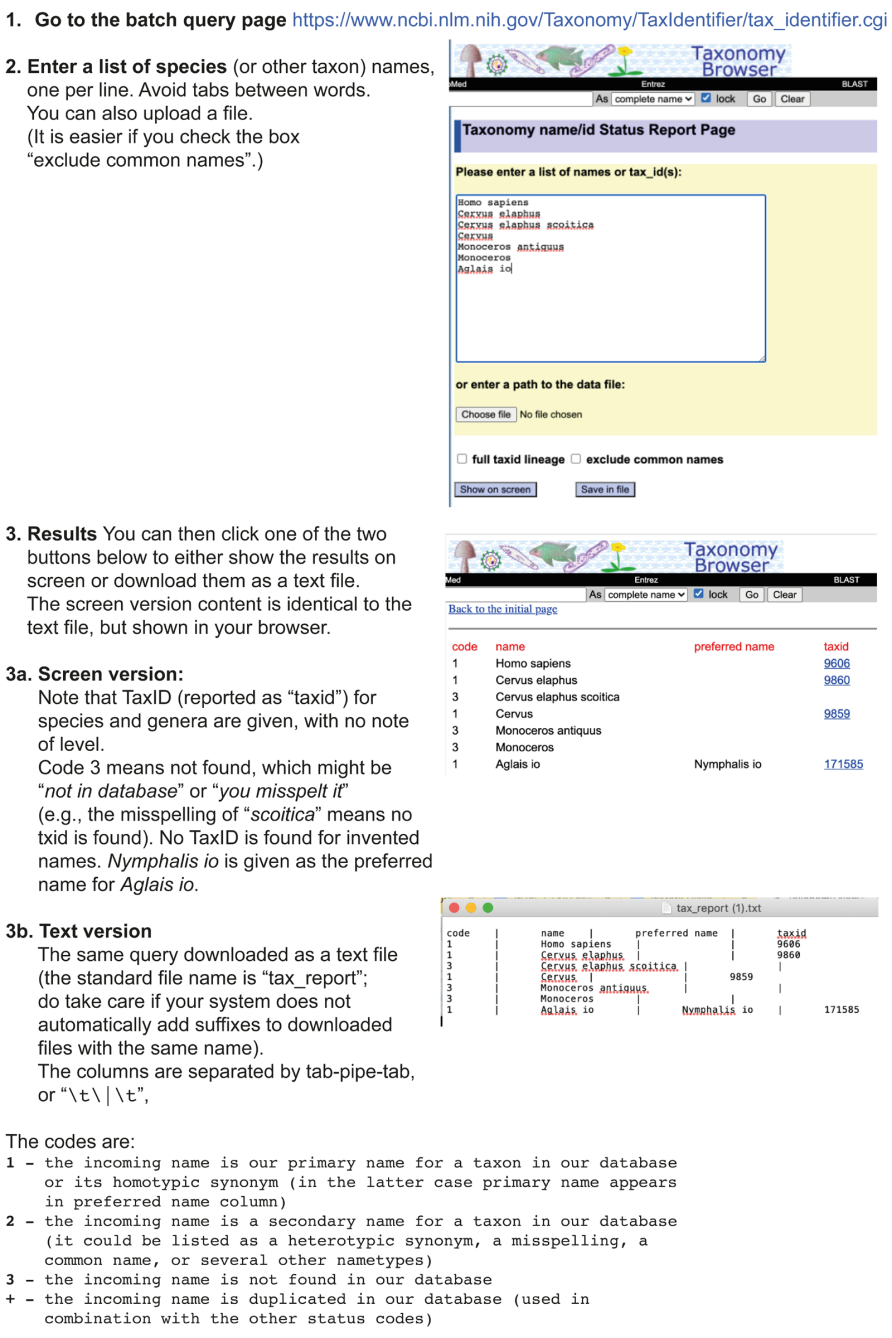
Using the NCBI Taxonomy web interface for bulk queries. Workflow illustrating the use of the NCBI Taxonomy web interface for bulk (multiple-name) queries, namely the batch query page https://www.ncbi.nlm.nih.gov/Taxonomy/TaxIdentifier/tax_identifier.cgi.

The NCBI web interface can be used to identify the correct TaxId for a single species of interest (workflow illustrated in Figure 3). If you have more than one or two species names for which you want to retrieve the TaxIds, TaxonomyDB provides a batch query Taxonomy Identifier form (workflow illustrated in Figure 4).

### Note: An updated NCBI taxonomy interface is being introduced

The NCBI Taxonomy displays are in the process of being updated at NCBI, with new displays and command line interface options. The new searchable interface is accessible through the following address: https://www.ncbi.nlm.nih.gov/datasets/taxonomy/tree/. Single, newly-designed pages with images and links to genomes and other resources are now available, e.g. https://www.ncbi.nlm.nih.gov/datasets/taxonomy/9860/. Download of taxonomic information can be done by clicking the download link in the detailed taxonomy names page: https://www.ncbi.nlm.nih.gov/datasets/taxonomy/9860/names/. The new command line interface allows queries to be run using NCBI Datasets tools in https://www.ncbi.nlm.nih.gov/datasets/docs/v2/how-tos/taxonomy/taxonomy/?utm_source=ncbi_insights&utm_medium=referral&utm_campaign=datasets-taxonomy-20240417. An initial blog post announced these new taxonomic interfaces ( https://ncbiinsights.ncbi.nlm.nih.gov/2024/04/17/browse-taxonomy-records-ncbi-datasets/), and a paper outlining further details is in preparation for the 2025 edition of the Nucleic Acids Research on databases. The legacy browser will remain accessible for a while and will be linked to from the new pages, but developments will only continue on the new version.

## Using the ENA Taxonomy service

**Figure 5.**
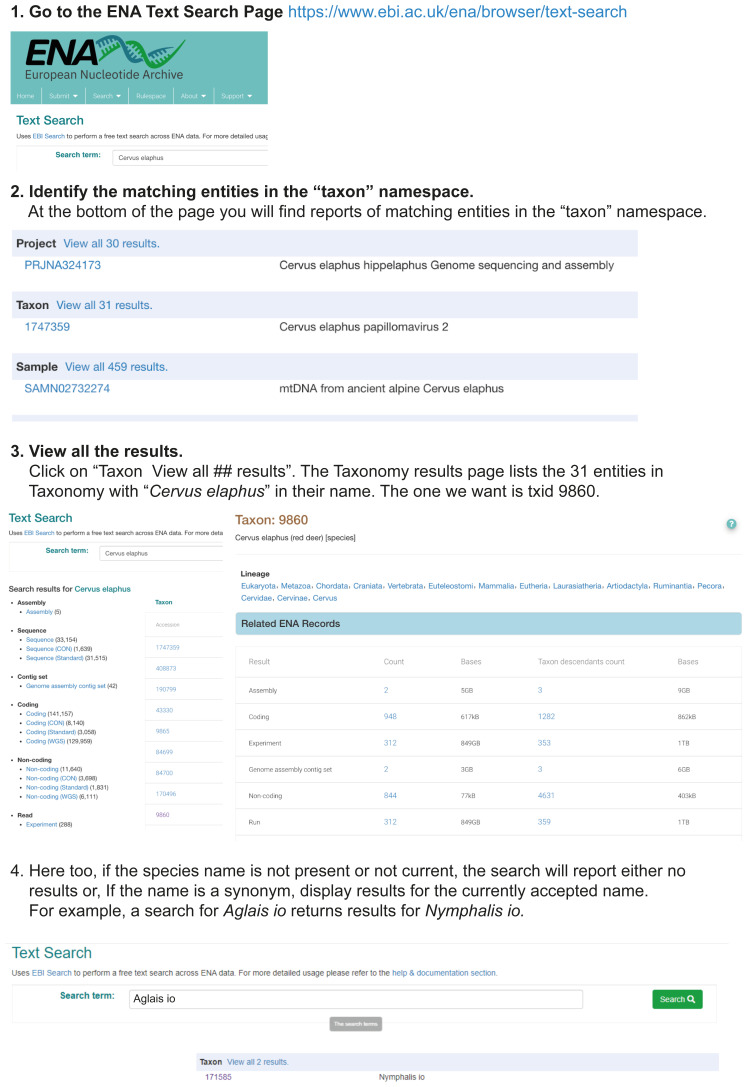
Using the ENA Text search interface for single queries. Workflow illustrating the use of the ENA web interface ( https://www.ebi.ac.uk/ena/browser/text-search) for a single-name query.

**Figure 6.**
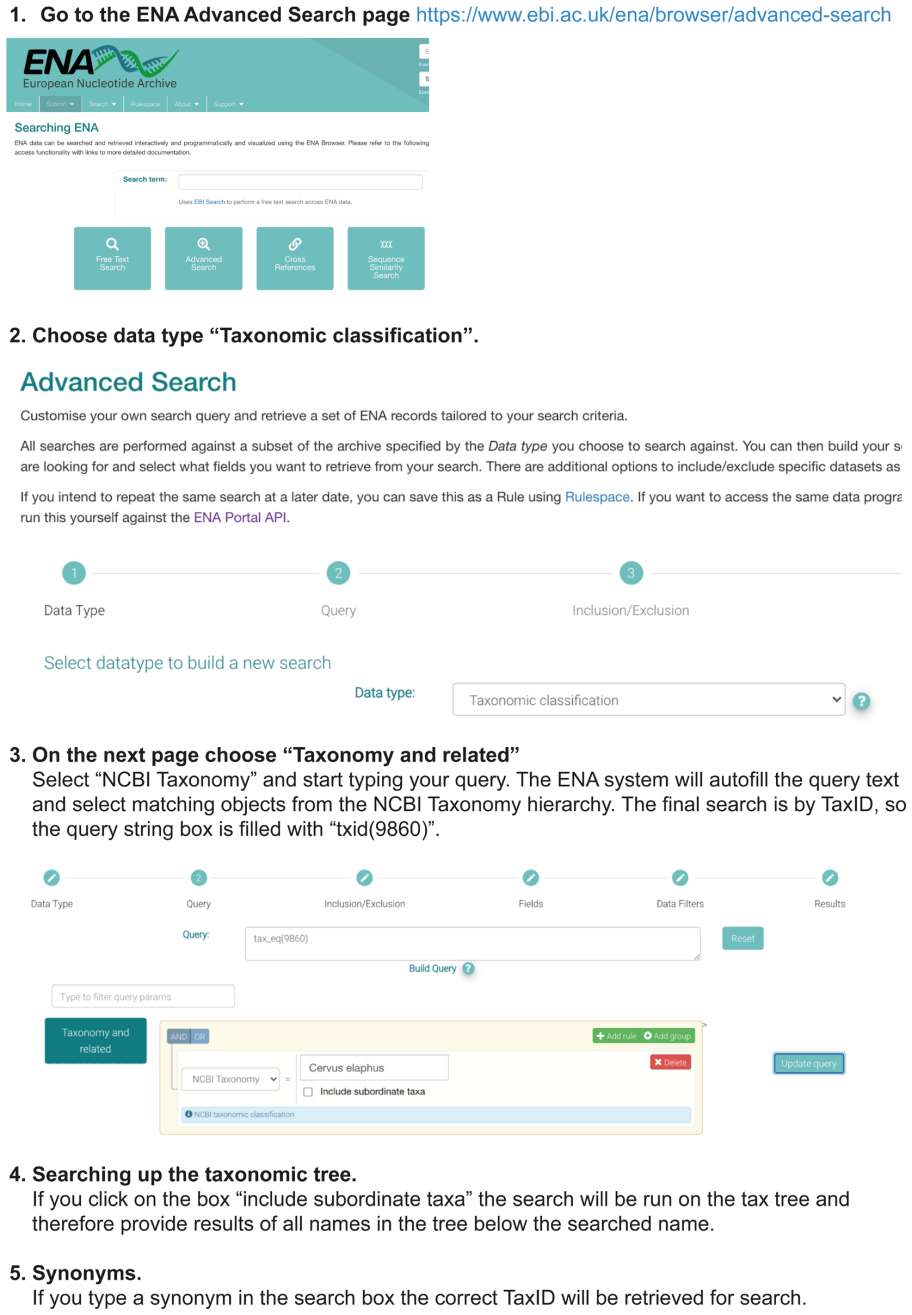
Using the ENA Advanced Search Interface for Single Queries. Workflow illustrating the use of the ENA advanced search web interface for a single-name query.

The ENA mirrors the NCBI Taxonomy, and thus it is also possible to perform searches in ENA for TaxIds corresponding to your target species. It is not as easy on the web, but the REST API is very useful. The workflows are demonstrated in Figure 5 and Figure 6.

### ENA Taxonomy service REST API Interface

The ENA REST API allows you to perform taxonomy searches automatically (and without the unusual logic that it is not the search that gives you the TaxId but the process of setting up the search). The API access mode also permits bulk queries. The interface is documented at https://www.ebi.ac.uk/ena/taxonomy/rest/#/. The ENA REST API displays any changes to the taxonomy made by the NCBI curators about 48 hours after the change has been made. The ENA REST API has several endpoints that allow searching differently on the taxonomy.

**Scientific name**

The scientific name search takes the form “ https://www.ebi.ac.uk/ena/taxonomy/rest/scientific-name/Species%20name”.

This search should be used if you know the scientific name of the organism (for example, to check after a name has been added to NCBI Taxonomy, that it is already available for submission and to get the TaxId). If you are unsure about the scientific name or you think that you might be searching for a synonym then you should use the “suggest for submission” tool. Please see below for details.

By sending an API query to ENA you will be able to retrieve the TaxId (noting that ENA uses the form “taxId” in reports) and some additional taxonomic information. You can do this in a standard web browser or from the command line or in programs using the “curl” command.

For example, for the red deer, *Cervus elaphus*, we submit an https query of the form:

https://www.ebi.ac.uk/ena/taxonomy/rest/scientific-name/Cervus%20elaphus

Note: the flag “scientific-name” is followed by a backslash “/” and the space in the name is changed to “%20”. If you are using a GUI browser, it is likely that the substitution of space for %20 will happen automatically. In curl requests, you must do the substitution before submitting: This works:

curl “ https://www.ebi.ac.uk/ena/taxonomy/rest/scientific-name/Cervus%20elaphus”

but the following does not work:

curl “ https://www.ebi.ac.uk/ena/taxonomy/rest/scientific-name/Cervus elaphus”

A successful query returns the following data:


  [ {
   “taxId” : “9860”,
    “scientificName” : “Cervus elaphus”,
    “commonName” : “red deer”,
    “formalName” : “true”,
    “rank” : “species”,
    “division” : “MAM”, 
    “lineage” : “Eukaryota; Metazoa; Chordata; Craniata; 
  Vertebrata; Euteleostomi; Mammalia; Eutheria; Laurasiatheria; 
  Artiodactyla; Ruminantia; Pecora; Cervidae; Cervinae; Cervus; “,
    “geneticCode” : “1”,
    “mitochondrialGeneticCode” : “2”,
    “submittable” : “true”,
    “binomial” : “true”
  } ]


From this we can check it is the correct taxon (a ruminant, yes) and learn two key things:

- taxId (the typographic form of TaxId used at ENA) is “ 9860”
- “ submittable” is “ true”; that is, we can submit genomic sequence data to this TaxId.

While most submittable-to TaxIDs are binomial species names, many subspecific names are able to have sequences submitted to them. For example, for *Cervus elaphus scoticus*:

curl “ https://www.ebi.ac.uk/ena/taxonomy/rest/scientific-name/Cervus%20elaphus%20scoticus”


   [ {
     “taxId” : “408873”,
     “scientificName” : “Cervus elaphus scoticus”,
     “commonName” : “Scottish red deer”,
     “formalName” : “true”,
     “rank” : “subspecies”,
     “division” : “MAM”,
     “lineage” : “Eukaryota; Metazoa; Chordata; Craniata; 
   Vertebrata; Euteleostomi; Mammalia; Eutheria; Laurasiatheria; 
   Artiodactyla; Ruminantia; Pecora; Cervidae; Cervinae; Cervus; “,
     “geneticCode” : “1”,
     “mitochondrialGeneticCode” : “2”,
     “submittable” : “true”,
     “binomial” : “true”
   } ]


Similarly, strains and hybrids are submittable-to. For example, there are data for a subspecies hybrid within *Cervus elaphus* (Cervus sp. 'elaphus sibiricus/xanthopygus' OG-2023).


[ {
  “taxId” : “3032040”,
  “scientificName” : “Cervus sp. 'elaphus sibiricus/xanthopygus' 
OG-2023”,
  “formalName” : “false”,
  “rank” : “species”,
  “division” : “MAM”,
  “lineage” : “Eukaryota; Metazoa; Chordata; Craniata; 
Vertebrata; Euteleostomi; Mammalia; Eutheria; Laurasiatheria; 
Artiodactyla; Ruminantia; Pecora; Cervidae; Cervinae; Cervus; “,
  “geneticCode” : “1”,
  “mitochondrialGeneticCode” : “2”,
  “submittable” : “true”,
  “binomial” : “true”
} ]


**Unsubmittable-to taxa**

If we ask for the TaxId of the genus *Cervus* we are told that *Cervus*, TaxId 9859, exists and is valid but cannot be submitted to.


[ {
  “taxId” : “9859”,
  “scientificName” : “Cervus”,
  “formalName” : “false”,
  “rank” : “genus”,
  “division” : “MAM”,
  “lineage” : “Eukaryota; Metazoa; Chordata; Craniata; 
Vertebrata; Euteleostomi; Mammalia; Eutheria; Laurasiatheria;
Artiodactyla; Ruminantia; Pecora; Cervidae; Cervinae; “,
  “geneticCode” : “1”,
  “mitochondrialGeneticCode” : “2”,
  “submittable” : “false”,
  “binomial” : “false”
} ]


Note that the placeholder “ *Cervus* sp.” is of species rank and submittable, but it is not binomial:


[ {
  “taxId” : “27598”,
  “scientificName” : “Cervus sp.”,
  “formalName” : “false”,
  “rank” : “species”,
  “division” : “MAM”,
  “lineage” : “Eukaryota; Metazoa; Chordata; Craniata;
Vertebrata; Euteleostomi; Mammalia; Eutheria; Laurasiatheria; 
Artiodactyla; Ruminantia; Pecora; Cervidae; Cervinae; Cervus; “,
  “geneticCode” : “1”,
  “mitochondrialGeneticCode” : “2”,
  “submittable” : “true”,
  “binomial” : “false”
} ]


The scientific name endpoint only provides results for the exact scientific name and does not include synonyms. If we misspell the name ( https://www.ebi.ac.uk/ena/taxonomy/rest/scientific-name/Cervus%20elapha) we get no results…


 [ ]


**Suggest for submission**

Another tool at ENA that may be helpful is the **suggest-for-submission** tool ( https://ena-docs.readthedocs.io/en/latest/faq/taxonomy.html?highlight=taxonomy). This is recommended in situations where you are unsure of the taxon name. This will provide all possible results that fit the search criteria.

In this case if we search for *Aglais io* (a homotypic synonym of *Nymphalis io* ( https://www.ebi.ac.uk/ena/taxonomy/rest/suggest-for-submission/aglais%20io), the datasystem reports:


[ {
  “TaxId” : “171585”,
  “scientificName” : “Nymphalis io”,
  “commonName” : “European peacock”,
  “displayName” : “Aglais io”
  “binomial”: “true”
} ]


Also, if we are interested in knowing the species available for submission within a given genus we can search for example for *Cervus*, and the system will report all available IDs. Extract from the response:


[
  {
    “taxId”: “9860”,
    “scientificName”: “Cervus elaphus”,
    “commonName”: “red deer”,
    “binomial”: “true”,
    “displayName”: “Cervus elaphus Linnaeus 1758”
  },
  {
    “taxId”: “9861”,
    “scientificName”: “Cervus canadensis canadensis”,
    “commonName”: “Eastern wapiti”,
    “binomial”: “true”,
    “displayName”: “Cervus elaphus canadensis Erxleben, 1777”
  },
  {
    “taxId”: “9863”,
    “scientificName”: “Cervus nippon”,
    “commonName”: “sika deer”,
    “binomial”: “true”,
    “displayName”: “Cervus nippon Temminck, 1838”
  },
…
]


**Search ENA by TaxId**

It is also possible to search ENA directly by a TaxId to confirm if it is submittable and binomial, using: https://www.ebi.ac.uk/ena/taxonomy/rest/tax-id/TaxId.

In situations where TaxIds have been previously merged, this tool redirects to the current TaxId and displays also the merged TaxId. For example, if we search for TaxId 257370 https://www.ebi.ac.uk/ena/taxonomy/rest/tax-id/257370, we will get:


[ {
  “taxId”: “257370”,
  “scientificName”: “Caligus elongatus”,
  “formalName”: “true”,
  “rank”: “species”,
  “division”: “INV”,
  “lineage”: “Eukaryota; Metazoa; Ecdysozoa; Arthropoda;
Crustacea; Multicrustacea; Hexanauplia; Copepoda;
Siphonostomatoida; Caligidae; Caligus; “,
  “geneticCode”: “1”,
  “mitochondrialGeneticCode”: “5”,
  “submittable”: “true”,
  “binomial”: “true”,
  “merged”: “[3109115]”
} ]


## Frequently asked questions

### Q1: The NCBI Taxonomy name for my species differs from the one I would like to use: what should I do?

**TL;DR: Please contact the NCBI Taxonomy helpdesk ( info@ncbi.nlm.nih.gov) to fix any issues you identify.**

If your chosen species name is the result of splitting or lumping, consult with NCBI Taxonomy to make the correct changes.

Taxonomy is a human endeavour, and thus the names of species can change. Formal changes in nomenclature are frequent, and community use of species names can take some time to follow best practice in the technical taxonomic literature.

Thus *Aglais io*, the European peacock butterfly, is also known as *Inachis io* and *Nymphalis io*. Linnaeus called it *Papilio io*. The current accepted name in the NCBI Taxonomy is *N. io*, and *A. io, P. io* and *I. io* are homotypic synonyms. See https://www.ncbi.nlm.nih.gov/Taxonomy/Browser/wwwtax.cgi?mode=Info&id=171585&lvl=3&lin=f&keep=1&srchmode=1&unlock

**My desired species name should be the current homotypic synonym.**

If you want to use a species name that you think is current and correct, but the same species concept is present in NCBI Taxonomy under what you believe is a junior homotypic synonym, then you should contact NCBI Taxonomy curators or liaise with the ENA helpdesk to request (or suggest) a change. You should simply provide proper reference for the change (e.g. the paper where the change was effected). The TaxId for the species is retained and just the binomen changed.

**My desired species name is a new name derived from splitting or lumping of previous names.**

If the species name you wish to use for your specimen is not in TaxonomyDB and was defined as part of splitting of a previous name through diagnosis of a previously cryptic group or merging of previous species concepts under a new name, again you should contact NCBI Taxonomy curators or liaise with the ENA helpdesk to request (or suggest) a change. You should simply provide proper reference for the change (e.g. the paper where the change was effected). This will result in a new TaxId being created for your species.

### Q2: The NCBI Taxonomy taxonomic hierarchy above the species level for my taxon is different from the one I would use. Is this a problem?

**TL;DR: This is not a problem for species TaxId, but please do work with NCBI Taxonomy to fix any deeper taxonomy issues.**

NCBI Taxonomy aspires to represent the taxonomic hierarchy for species, genera, families, etc as correctly as possible. However, the NCBI Taxonomy team can only keep up with revisions and changes if kept informed by an engaged community, and cannot represent alternate, conflicting hierarchies. For the taxonomy above the generic level, NCBI Taxonomy relies on experts (or teams of experts), and they welcome engagement.

If you think that the taxonomy tree as represented in NCBI Taxonomy is out of date (or plain wrong), please do contact them, or liaise with the ENA data curators. You should provide proper reference for the different hierarchy (e.g. the paper where the new proposal was made). They may:

- Immediately update the tree to represent the new model you present
- Suggest you consult with their domain experts to resolve whatever issues you have with the hierarchy.

The absolute truth of the hierarchy does not affect the species name, and any change in the hierarchy above the genus level will simply bring the child taxa (genera, species and subspecific taxa) along with it. If the proposal implies moving genera between families (or other subfamilial taxa), then again the species and subspecific taxa will also move. Thus “fixing” the NCBI Taxonomy hierarchy should not delay acquisition of a TaxId for a species or species-level taxon.

### Q3: I want to submit data for a species, but it is not in the NCBI Taxonomy. How do i get it into the NCBI Taxonomy?

If you need to add a species that is not in NCBI Taxonomy, you should contact NCBI taxonomy curators through info@ncbi.nlm.nih.gov or the ENA helpdesk through the Register novel taxonomy form in the Webin Submission Portal ( https://www.ebi.ac.uk/ena/submit/webin/login).

To make the request you will need the list of taxa to be added, their placement in the taxonomy (at NCBI) and a reference for the formal naming of the taxa. Requests can be made individually, but if several taxa are requested at the same time, the information should be sent in a spreadsheet. The following information should be included (a template spreadsheet can also be found in the ENA Webin Portal in the “Register taxonomy” option). The ENA team will liaise with NCBI to generate the TaxId for each taxon as requested. Adding new taxa and issuing new TaxId takes a few days, so please initiate this process as soon as you realise it is needed.

Please see https://ena-docs.readthedocs.io/en/latest/faq/taxonomy_requests.html for more details.

### Q4: I have a strain, isolate or specimen for which i do not (yet) have a species ID. How do I assign a TaxId to it?

If you have strain, isolate or specimen for which you do not yet have a species ID, you can request a TaxId to be generated for an informal name that will act as a placeholder for the taxon. The informal names requested need to follow the guidelines for submittable organism names (see https://ena-docs.readthedocs.io/en/latest/faq/taxonomy_requests.html).

Please use the format described in FAQ3 above to submit your proposed names to ENA for consideration. If several taxa are requested at the same time, the information should be sent in a spreadsheet formatted as in Table 1. The ENA team will liaise with NCBI to generate the TaxId for each taxon as requested.

**Table 1.** Spreadsheet format for submission of proposed names to ENA.

*Column*	*Name*	*Content*
*1*	proposed_name	the organism name (mandatory). ENA will check if there is a taxon already registered with the given name.
*2*	name_type	allowed taxon name types are Environmental Name Synthetic Name Novel Species Unidentified Species Published Name
*3*	host	host associated with the taxon, if applicable
*4*	project_id	project associated with the taxa, if applicable
*5*	description	a short description of the taxon, please provide an authority or publication where available, or any other information describing the organism

**Updating informal names with full species names.**

When a registered specimen, isolate or strain has been identified to species, and (if it is a new species) the name has been formally published, the TaxId can be updated in NCBI Taxonomy. This could involve simply moving the strain TaxId to be a child of an existing registered species, or adding the new species to NCBI Taxonomy.

You can do this by contacting NCBI TaxonomyDB curators or liaising with the ENA helpdesk and sending the published name and link to the publication. Note that (as with other forms of homotypic synonymy) the TaxId will remain the same, but the record will be updated in NCBI Taxonomy.

### Q5: What is a ToLID?

Tree of Life Identifiers (ToLIDs) are a system for naming individual specimens to track them through the sequencing process and to act as assembly *Names* when the data are submitted to the INSDC databases. The ToLID system is described at https://id.tol.sanger.ac.uk/. ToLID has been adopted as the identifier-of-choice for the Earth BioGenome Project and it is strongly recommended that specimens have ToLIDs assigned as early in the process of genomic sequencing as is possible. ToLIDs can be added to the Biosample record for a specimen, and provide a useful and unique naming system to avoid namespace clashes and reduce confusion. ToLIDs are constructed from two letters indicating the general area of the taxonomy the species derives from ( *il* indicating Insecta, Lepidoptera) and then seven letters derived from the species binomen ( *AriAgre* for *Aricia agrestis*) and then a number that increments for each specimen added. Over 480,000 taxa in the NCBI Taxonomy have been allocated unique xyGenSpec TaxId names, with potential clashes dealt with. For historical reasons, the ToLIDs for vertebrates differ in consisting of a one letter prefix and a six letter species binomen abbreviation.

New taxa added to NCBI Taxonomy are allocated ToLIDs as they are made public.

Species names can change, but ToLIDs will not. For example, if a specimen is allocated a ToLID of mHomSap and the species name is later revised to *Homo stupidus* or *Pan sapiens*, the ToLID of specimens acquired to date will remain the same. This constancy avoids having to propagate changes through many systems but can result in confusing apparent mis-namings. However, because the ToLID registry only emits unique ToLIDs, the system retains the specimen-uniqueness required to track individual organisms through multiple analyses and processes. If a specimen is mis-identified, we encourage users of ToLID to request a new ToLID reflecting the changed information.

See the ToLID website at https://id.tol.sanger.ac.uk/ for instructions on requesting ToLIDs for your specimens. To qualify, a specimen needs a TaxId where “submittable = true” and “binomial = true”.

Please note that ToLIDs are assigned at species level. It is possible to request ToLIDs at infra-species levels (e.g. subspecies, variant), but the resulting ToLID will be based on the parent species prefix. If your specimen consists of a pool of individuals (e.g. because they are clonal, of small size or such) you should register the pool as one ToLID. A distinct pool should be registered as another ToLID.

### Further reading: ENA FAQ concerning Taxonomy Requests

ENA’s continuously updated frequently asked questions pages are the best resource for any queries about their systems: see https://ena-docs.readthedocs.io/en/latest/faq/taxonomy_requests.html
