# Supplementary material for: Taxonomy Identifiers (TaxId) for Biodiversity Genomics: a guide to getting TaxId for submission of data to public databases
Source: Wellcome Open Res. 2024 Oct 15;9:591. [Version 1] doi: 10.12688/wellcomeopenres.22949.1 (PMC11544195; doi:10.12688/wellcomeopenres.22949.1)
Supplement: Peer review history

# Reviewer report: Reviewer response for version 1

**DOI:** 10.21956/wellcomeopenres.25270.r107160

**Published:** 2024-12-08

**Authors:** Virginia Valcárcel

**Affiliations:** 1 Universidad Autonoma de Madrid, Madrid, Community of Madrid, Spain

The guidance provided is sound and timely. The submission of molecular data to NCBI and ENA is an ongoing activity that is growing exponentially, and the use of proper taxonomic identification of the organism is key to making these online databases useful to the entire scientific community. By providing a thorough explanation of the process, this guide allows users to understand the taxonomic system behind these databases and facilitates correct submissions from a taxonomic point of view.

 I have general suggestion about the structure, and that is why I said "Partly" to the question "Are sufficient details provided to allow replication of the method development and its use by others?". Also the reason why I said "no" to the question "Are the conclusions about the method and its performance adequately supported by the findings presented in the article?" Is because there are no conclusions, but this kind of publication does not need for conclusions. The answer to this question that would reflect my opinion is "not applicable".

 Finally, I have minor comments on some typos that I list at the bottom.

**Regarding the structure:**

 I am not familiar with this type of publication, but I often use FAQS and online resources to guide submissions, and I find that the structure of this guide is not easy to follow. Here are the things I would change about the structure:

1. I would put a section about what a TOLID is right after "What is a TaxId and what kind of TaxId do I need?
2. I would move the heading "TaxId currency" after the description of how to do the searches.  
3. In "Finding out if my species has a TaxId" I would include a brief description that there are different search systems and the main differences. For example, I am not quite sure what you mean by "NCBI API system" and where you describe it in the guide, because you say "Do single or bulk queries using the NCBI API (see below)", but then there is no easily identifiable heading that refers to this. There is a heading to search NCBI via the web and then the ENA API system, is this the same as the NCBI API system? Related to this, I do not fully understand the paragraph related to the NCBI taxonomy displays that are currently being updated, I suggest that you include a subheading and briefly introduce the purpose of including this information, why it is useful to users.
4. I would replace "Unsubmittable-to taxa" with another heading that gives more information, such as "How to submit when the species is unknown". Then I would start by saying that only binomials are submittable-to taxa, so you cannot submit to genera. However, if the data are from a species that has not yet been described, you can submit to "genus sp". And then continue with all the explanations you provide in this section.
5. I also don't understand "Suggest for submission" - is this a specific search system? Or is this just to indicate that you can use an outdated taxonomy and the search will still work and retrieve the correct name of the taxa according to the updated taxonomy? For me there are two different things here, one is to convey the idea that you can search for outdated names, and the other is that if you are not sure of the name of the species, you can search for the genus and then get the TAXIDs for all the species/subspecies/varieties within it. I will separate the two.
6. I suggest that you divide the **Using the ENA Taxonomy service **heading in two parts:

- One based on how to do searches to make taxonomic queries. I would divide this into two subheadings:
  i) One to explain how to search when you do not know the TaxID. Here I would include the notation - and note here that you can also search for outdated names here - how to search if you are not sure about the species name (search for the genus).
  ii) how to do it when you know the TAxID.
- The other one is to describe all the issues related to submitting, what are submitted-to taxa and here include the idea that if the species is yet to be described then you can submit by using the name of the genus and adding sp. afterwards.
  7. The names in the subheadings of "Using the ENA Taxonomy Service" are not clear to me. For example, would it be correct to say "How to perform automatic searches in ENA?" instead of "ENA Taxonomy Service REST API Interface"?
  8. Also, I would provide more information for "Scientific name" as it is now, the reader wouldn't know if you are going to describe how to search scientific names or if you are going to provide information about the ENA taxonomy... this heading is a bit too vague for me. Also, I wouldn't start this section with this: *"The scientific name search takes the form “https://www.ebi.ac.uk/ena/taxonomy/rest/scientific-name/Species%20* *name”.* Instead, I would first describe the notation and include all the cases (species and infraspecies together, something like this:

“To submit a http query be aware that the flag “scientific-name” must be followed by a backslash “/” and the spaces between names must be replaced by “%20”. If you are using a GUI browser, it is likely that the substitution of space for %20 will happen automatically. In curl requests, you must do the substitution before submitting.

 For example, to submit a https query for *Cervus elaphus *this will work:

 curl “ https://www.ebi.ac.uk/ena/taxonomy/rest/scientific-name/Cervus%20elaphus”

 but the following does not work:

 curl “ https://www.ebi.ac.uk/ena/taxonomy/rest/scientific-name/Cervus elaphus”

 Also, if you want to submit a http query for any taxa below species, you must include the scientific names without any reference to the taxonomic rank (that is, only three words) and replace all spaces by for %20.

 For example, to submit a https query for *Cervus elaphus *subsp. *scoticus* this will work:

 curl “ https://www.ebi.ac.uk/ena/taxonomy/rest/scientific-name/Cervus%20elaphus%20scoticus”

**Minor comments:**

1. Introduction,
  i) I would replace “attribution” by “identification”
  ii) when you say “ *to every* *node in the tree of life.* *” *You mean to every node that corresponds to a taxon rank (family, genus, species…), right? I would specify it.
2. By “Senior synonym” you mean the correct (i.e., accepted) name for a species concept? To me this is not a synonym. Synonyms are valid names that are not recognized for a variety of reasons, but the accepted name is not a synonym to me. But I’m not an expert on nomenclature.
3. Replace “species” by “taxon” in the heading “Finding out if my species has a TaxId”
4. In this sentence "the NCBI web interface can be used to identify the correct TaxId for a single species of interest" you are referring to species, but you can also search for subspecies, right? Then I suggest that you either indicate at the beginning of the guide that when you refer to species you are referring to species and all taxonomic ranks below, or that every time you refer to species/subspecies/varieties... you specify it.
5. I would change this:  “It is also possible to search ENA directly by a TaxId to confirm if it is submittable and binomial, using: https://www.ebi.ac.uk/ena/taxonomy/rest/tax-id/TaxId.” To make explicit in some way that after typing https://www.ebi.ac.uk/ena/taxonomy/rest/tax-id/ you need to type the desired TaxId.
6. In Q4, capitalize the second “I”: *Q4: I have a strain, isolate or specimen for which I do not (yet) have a species ID. How do I assign* *a TaxId to it.*

Is the rationale for developing the new method (or application) clearly explained?

Yes

Is the description of the method technically sound?

Yes

Are the conclusions about the method and its performance adequately supported by the findings presented in the article?

No

If any results are presented, are all the source data underlying the results available to ensure full reproducibility?

No source data required

Are sufficient details provided to allow replication of the method development and its use by others?

Partly

Reviewer Expertise:

phylogeny, taxonomy, plants, ecology

I confirm that I have read this submission and believe that I have an appropriate level of expertise to confirm that it is of an acceptable scientific standard, however I have significant reservations, as outlined above.

# Reviewer report: Reviewer response for version 1

**DOI:** 10.21956/wellcomeopenres.25270.r105550

**Published:** 2024-11-08

**Authors:** Ole Kristian Tørresen

**Affiliations:** 1 University of Oslo, Oslo, Norway

The authors describe the rational behind the taxonomic identifier from NCBI, and the process of obtaining one for a species of interest. Maybe most importantly, the process around how to create new taxonomic identifiers is discussed. Related matters such as ToLID is also mentioned.

 It is good to have a detailed description of the process around taxonomic assignment such as this. Many times it is difficult to figure out what actually goes on “under the hood”. The information here might have been available before, but maybe spread across multiple sources. Therefore it is essential to have one place to go to when needing this information. 

 The article is less formal than what one might expect from a scientific publication. This is not necessarily a bad thing, but it might put some readers off. For instance the TL; DR parts. The structure is also a bit different.

 There is something off in the second sentence of the abstract. Something missing?

Is the rationale for developing the new method (or application) clearly explained?

Yes

Is the description of the method technically sound?

Yes

Are the conclusions about the method and its performance adequately supported by the findings presented in the article?

Yes

If any results are presented, are all the source data underlying the results available to ensure full reproducibility?

Yes

Are sufficient details provided to allow replication of the method development and its use by others?

Yes

Reviewer Expertise:

(Comparative) Genomics, assembly, annotation

I confirm that I have read this submission and believe that I have an appropriate level of expertise to confirm that it is of an acceptable scientific standard.

# Reviewer report: Reviewer response for version 1

**DOI:** 10.21956/wellcomeopenres.25270.r107147

**Published:** 2024-11-07

**Authors:** Henrik Nilsson, Arnold Tobias Jansson

**Affiliations:** 1 University of Gothenburg, Gothenburg, Sweden

**Review of Blaxter et al., ”Taxonomy Identifiers…”**

 We have now been through the manuscript of Blaxter et al. It is a hands-on guide for getting TaxID for submission of data to public databases of the INSDC family. (In that sense, maybe the title overpromises a bit. After all, the concept of “public databases” encompasses many databases indeed (such as Wikipedia and IMBd), only a tiny proportion of which use the TaxID concept. Maybe “public databases” should be qualified in the title?) We are in favour of illustrated guides of this kind, and we imagine that this guide will prove helpful to many INSDC users. The manuscript is commendably illustrated.

 Ideally, the manuscript would have sported line numbers, which would have made our comments easier for the authors to pinpoint.

**Abstract**

 The sentence “Tracking of…” does not look complete. Is a verb missing?

 “resolving taxonomic issues” is likely to mean very different things to different readers. Should it be qualified here to give the reader a feeling for what these could be? “resolving taxonomic issues such as XXX” ?

 Further up in the abstract, the authors qualify the INSDC as NCBI, ENA, and DDBJ. However, in the sentence “Discuss directly” as well as in the remainder of the manuscript, DDBJ is not mentioned. This could mean many things. One is that DDBJ does not use the TaxID concept. Another is that DDBJ uses it, but that they are not actively involved in its development and thus cannot offer support. Does it make sense to indicate the reason why in the manuscript? Otherwise, this matter is open to interpretation.

**Introduction**

 “all Earth’s” > “all of Earth’s”

 It is perhaps not fully correct to claim that the nomenclatural codes offer a “complete solution to the naming of species”. For fungi, the “botanical” code requires a type specimen, which excludes large numbers of (so-called “dark”) fungi from formal description. A nomenclatural code that imposes such paraphyletic restrictions on organism groups should probably not be called “complete”.

 The situation is the same with prokaryotes. Speaking of which, the manuscript is surprisingly silent on prokaryotes and viruses. Is the TaxID concept not applicable to them, some readers are likely to ask.

 The manuscript is also silent on the exact conditions under which species/taxon names can be introduced for TaxID acquisition. Species/taxon names introduced under the SeqCode, for instance, can they get TaxIDs? Or species/taxon names that are not validly published but that still correspond to monophyletic units in nature and that see frequent use in the literature, what about them? (e.g., https://mycokeys.pensoft.net/articles.php?id=125549&journal_name=mycokeys). It probably makes sense to be clear on what kind of names that qualify for TaxID acquisition.

 “for animals, plants, fungi, and protists”. Do the authors simply mean “eukaryotes” here? If so, it is probably better to write that. The present wording is open to the interpretation that, say, myxomycetes, oomycetes, and algae are not targeted or covered in this “system”.

 “data, and gives” > “data and gives” ?

 “from Kingdom” > “from kingdom”

**What is a TaxID and…**

 The statement “You need the TaxID of a species (or a name below species level) to submit sequence data” is perhaps a bit misleading. It gets it full explanation only on page 13, “Note that the placeholder…”. The way it is now, the sentence may be taken to mean sequences can be submitted only for taxa identified to species level in the sense of a Latin binomial. There is some risk that some readers will not understand that the authors intend “Nematoda sp.” as a species name. Why not move the explanation from page 13 to this page to remove this uncertainty?

 It is perhaps not entirely clear what is intended with “cellular life”. Is there life that is not “cellular”? Or is this to distinguish cellular life from viruses? But viruses are in NCBI taxonomy which would imply that other rules apply for them. In fact, viruses are completely left out from the manuscript.

 On page 6 we read that the “environmental sample” flag should be used for “metagenomic and metabarcoding samples”. But there will be many examples of “environmental samples” that are indeed environmental samples, but that are neither “metagenomic” nor “metabarcoding samples”. A Sanger sequence produce from a soil sample, for instance, or from an unknown plant leaf. A more precise definition of “environmental sample” would probably be good here. Are HTS-derived sequences intended?

 “DDBJ, ENA and NCBI” – the manuscript sometimes uses the Oxford comma (such as in the abstract), sometimes not (such as here). Would it make sense to streamline this in the manuscript?

 In the final paragraph of this section, the authors invite readers and users to contact the helpdesk in case of problems. That’s a nice thing to do, and we’re all for it. However, exactly how you contact the help desk is only mentioned on page 15. Would it make sense to mention it here too, or here instead?

**TaxID currency**

 The authors used the term “portmanteau” on page 4 to indicate that some names are placeholders for many distinct species. “Nematoda sp.” would be such an example, as would the commonly used “Uncultured fungus”. But this means that not all distinct species in the INSDC will have a unique TaxID, since many distinct species are lumped together under placeholder names. “for all species in INSDC” should probably be revised to “for all species names in INSDC” as a consequence.

 “on databases” > “database issue”.

 The statement “without the unusual logic” comes across as too indirect and difficult to understand. Can it be simplified?

 “or, If the name” > “or, if the name”

 The wording “on the tax tree” is perhaps not crystal clear. But this is the “taxonomic tree” that the authors mention on page 4, correct? If so, maybe it is better to write “taxonomic tree” here too.

 “check it is” > “check whether it is” ?

**Frequently asked questions**

 The statement “The absolute truth of the hierarchy…” is surprising and hard to interpret. When is a hierarchy “true”? Can a hierarchy be false? We imagine that the authors simply mean “The degree to which the hierarchy is up-to-date with respect to the latest research results…”. If so, then please consider writing that (or something along that line) instead.

 “any other information describing the organism” – The word “describing” is possibly a bit unfortunate here. The statement can be read to refer to formal description of species and higher groups. We don’t think that’s the intended interpretation here, however. What about “characterizing” instead? But if indeed formal description is intended, then please be explicit about it.

 “e.g. subspecies, variant” > “e.g. subspecies and variant” ?

Is the rationale for developing the new method (or application) clearly explained?

Yes

Is the description of the method technically sound?

Yes

Are the conclusions about the method and its performance adequately supported by the findings presented in the article?

Yes

If any results are presented, are all the source data underlying the results available to ensure full reproducibility?

Yes

Are sufficient details provided to allow replication of the method development and its use by others?

Yes

Reviewer Expertise:

Systematics, taxonomy, mycology, bioinformatics, biodiversity informatics

We confirm that we have read this submission and believe that we have an appropriate level of expertise to confirm that it is of an acceptable scientific standard.

## References

[1] ﻿Phylogenetic classification of arbuscular mycorrhizal fungi: new species and higher-ranking taxa in Glomeromycota and Mucoromycota (class Endogonomycetes). Reference source 10.3897/mycokeys.107.125549PMC1133639639169987

# Reviewer report: Reviewer response for version 1

**DOI:** 10.21956/wellcomeopenres.25270.r107161

**Published:** 2024-11-01

**Authors:** Emilio Berti

**Affiliations:** 1 German Centre for Integrative Biodiversity Research (iDiv) Halle-Jena-Leipzig, Leipzig, Saxony, Germany

I have read the paper titled "Taxonomy Identifiers (TaxId) for Biodiversity Genomics: a guide to getting TaxId for submission of data to public databases" with great interest. This paper explains how to retrieve the NCBI TaxId for a species, if it exists, using web interfaces or command line (e.g., curl) tools. In addition, it explains how to request a new TaxId if the species was not registered before in the NCBI database. Moreover, this paper elucidate some aspects on how the NCBI and the ENA databases interact, which I found very well explained and particularly useful for users of ENA.

 I was quite surprised that the technical guidelines provided by this paper were not published earlier, as their goal is not only important, but necessary for experiments to deposit their data. This paper is a simple, yet effective guide that can be useful to many researchers.

 The manuscript is clear and very well written, the figures are relevant and well presented. I liked the choice of showing the API functionality through simple programs (e.g., curls), instead of the many packages that exist for R and python: because this study aims at providing a guide for submission of genomic data, using advanced programming packages does not seem particularly relevant.

 I have no major or minor suggestions for the study, except that I could not understand the second sentence of the abstract. Is a verb missing?

 Disclaimer: I am familiar with taxonomic challenges in Ecology, but not in genomic research.

Is the rationale for developing the new method (or application) clearly explained?

Yes

Is the description of the method technically sound?

Yes

Are the conclusions about the method and its performance adequately supported by the findings presented in the article?

Yes

If any results are presented, are all the source data underlying the results available to ensure full reproducibility?

No source data required

Are sufficient details provided to allow replication of the method development and its use by others?

Yes

Reviewer Expertise:

ecology, biogeography, macroecology

I confirm that I have read this submission and believe that I have an appropriate level of expertise to confirm that it is of an acceptable scientific standard.
